# Supplementary material for: Recollection in the human hippocampal-entorhinal cell circuitry
Source: Nat Commun. 2019 Apr 3;10:1503. doi: 10.1038/s41467-019-09558-3 (PMC6447634; doi:10.1038/s41467-019-09558-3)
Supplement: Supplementary file 1 — Supplementary Information [file 41467_2019_9558_MOESM1_ESM.pdf]

Supplementary Information

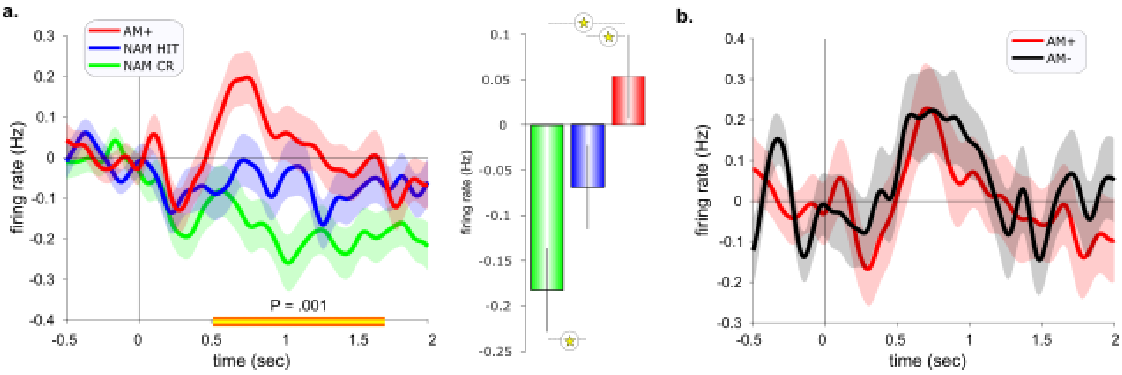

Supplementary Figure 1. Entorhinal cortex (EC) engagement during successful associative memory retrieval (AM+). **a.** Mean EC firing rates across neurons (n=211, baseline-corrected) during successful associative retrieval (AM+), successful recognition of old images (NAM HIT) and successful identification of new images (NAM CR). Shaded areas show average standard error across all pairwise condition differences. Horizontal yellow line indicates a significant cluster of repeated-measures ANOVA Condition effects from 500-1700 ms post stimulus onset (corrected for multiple comparisons across the entire 0-2000 ms time window). The cluster P value is .001 and the maximum effect size ($F_{(2,420)}$) is 15.88 at 768 ms. Bar plot shows results from pairwise follow-up contrasts, revealing a significant difference for all pairwise comparisons (AM+ vs. NAM HIT: $t_{(210)}$ = 2.66, P = .008; AM+ vs. NAM CR: $t_{(210)}$ = 4.47, P < .001; NAM HIT vs. NAM CR: $t_{(210)}$ = 2.67, P = .008). **b.** Mean EC firing rates across neurons (n=167, after removing participants with less than 10 AM- trials) during successful vs. unsuccessful associative retrieval (AM+ vs. AM-). Shaded areas show average standard error across condition differences. No statistical difference was observed between the two conditions.

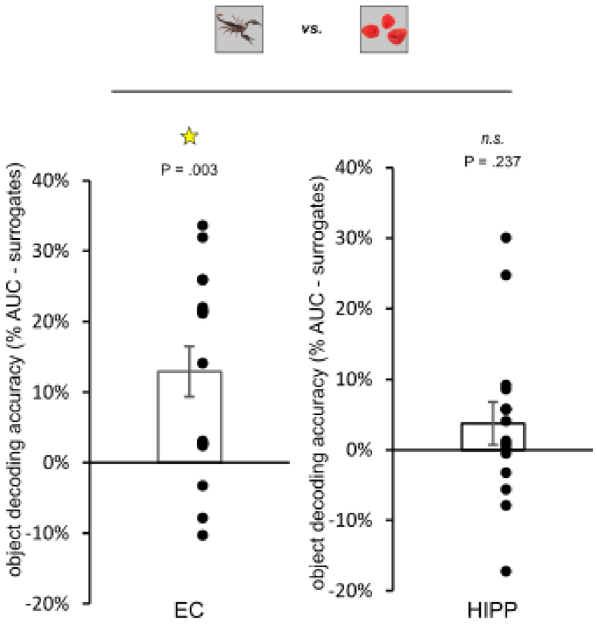

Supplementary Figure 2. Object decoding during AM encoding trials based on population codes. Results from 5-fold cross-validated LDA decoding for Entorhinal Cortex (EC, left) and Hippocampus (HIPP, right), based on spike trains averaged across the 3-sec stimulus presentation period. Bars show mean decoding accuracy ± SEM across participants (n=16 for EC, n=15 for HIPP) after subtracting the surrogate performance. Dots represent individual participants. One-sample t tests showed significant above-chance decoding accuracy of objects in EC ($t_{(15)}$ = 3.62, P = .003), but not in HIPP ($t_{(14)}$ = 1.24, P = .237).

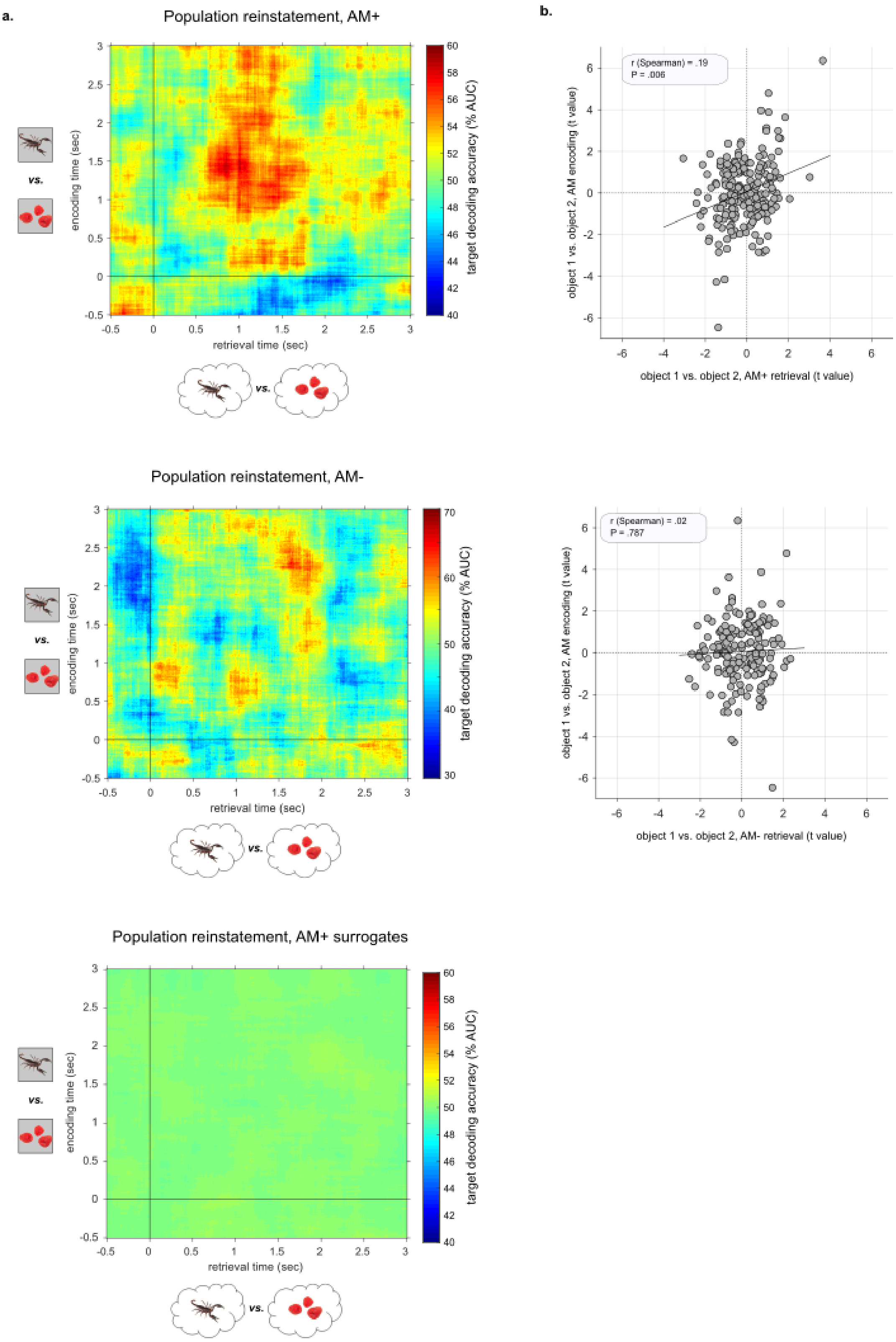

Supplementary Figure 3: **Memory-driven object reinstatement in entorhinal cortex. a.** Encoding time x retrieval time reinstatement maps used for the contrast maps in main Figure 3. *top*: AM+. *middle*: AM-; *bottom*: AM+ surrogates derived from shuffling the training labels 100 times per participant. **b.** Target object reinstatement across EC neurons. Top scatterplot is taken from main Figure 3c. Bottom scatterplot shows the same analysis for AM- trials.
